# A preoperative scoring system to predict the risk of No.10 lymph node metastasis for advanced upper gastric cancer: a large case report based on a single-center study

**DOI:** 10.18632/oncotarget.17273

**Published:** 2017-04-20

**Authors:** Zhi-Liang Hong, Qi-Yue Chen, Chao-Hui Zheng, Ping Li, Jian-Wei Xie, Jia-Bin Wang, Jian-Xian Lin, Jun Lu, Long-Long Cao, Mi Lin, Ru-Hong Tu, Chang-Ming Huang

**Affiliations:** ^1^ Department of Gastric Surgery, Fujian Medical University Union Hospital, Fuzhou 350001, Fujian Province, China

**Keywords:** stomach cancer, laparoscopy, spleen-preserving SHLND, No.10 LN metastasis, scoring system

## Abstract

**Purpose:**

To investigate upper stomach carcinoma risk factors for No. 10 lymph node (LN) metastasis, and establish a preoperative scoring system to predict No.10 LN metastasis.

**Method:**

Between January 2011 and December 2014, we prospectively collected and retrospectively analyzed the data of 398 patients with upper-third gastric cancer (GC) who underwent laparoscopic spleen-preserving hilar lymph-node dissection (SHLND). We use the logistics regression analysis risk factors of No. 10 LN metastasis to establish and verify a scoring model.

**Result:**

Among the 398 patients examined, 38 patients had No. 10 LN metastasis, yielding a 9.6% transfer rate. The preoperative risk factor analysis for No. 10 LN metastasis in the modeling group showed that tumor size, preoperative T staging, and preoperative N staging are independent risk factors. To establish a scoring system, we divided the modeling group of patients into three levels: low risk, intermediate risk, and high risk. The No. 10 LN metastasis rates of the low risk, intermediate risk and high risk groups were 2.84%, 13.9% and 34.9% respectively, with statistically significant (P < 0.001). The value for the area under the ROC curve of the scoring system was 0.820, and there were no statistically significant differences between the observed and predicted incidence rates for No. 10 LN metastasis in the validation set (P > 0.05).

**Conclusion:**

The scoring system comprising the tumor size, preoperative T stage and N stage is a simple and effective method to predict the risk of No. 10 LN metastasis and to preoperatively select cases suitable for laparoscopic spleen-preserving SHLND.

## INTRODUCTION

For advanced proximal gastric cancer (GC), the No. 10 lymph node (LN) is a crucial link in lymphatic drainage. Previous reports have found that the No. 10 LN metastasis rate is approximately 9.5% to 27.9% [1–3]. According to 14th edition of the Japanese gastric cancer treatment guidelines, D2 lymphadenectomy is the standard procedure for advanced GC, and the No. 10 LN should be dissected for the treatment of advanced upper GC. In recent years, as the concept of preserving viscera function and the use of minimally invasive technology has been accepted by an increasing number of clinicians, laparoscopic spleen-preserving splenic hilar LN dissection (SHLND) has become a valuable treatment option, and its use has gradually increased. In 2008, the South Korean scholar Hyung et al. [4] reported the first use of laparoscopic treatment to preserve the spleen during SHLND of upper GC, achieving a good curative effect and indicating that the operation is safe and feasible. Since then, the use of laparoscopic surgery to preserve the spleen during SHLND has increased [5]. However, this technology is difficult to implement in clinical practice because the spleen is deep, the operating space is narrow, and the splenic vessels are rich in this area and their branches are particularly complex. Thus, improvements in the preoperative prediction of No. 10 LN metastasis, which could provide medical evidence for SHLND indications, are urgently needed. However, few studies have focused on this topic. Therefore, we performed a retrospective study of patients subjected to laparoscopic spleen-preserving SHLND surgery to explore the preoperative factors associated with No. 10 LN metastasis and establish a new scoring system to preoperatively predict the risk of No. 10 LN metastasis.

## RESULTS

### Comparison of the clinicopathological characteristics of the patients in the model development and validation groups

The 299 patients in the model development group included 228 males (76.5%) and 70 females (23.5%) with a mean age of 60.44 ± 10.29 years. The average body mass index (BMI) of the patients was 22.15 ± 2.71 kg/m^2^. The preoperative clinicopathological characteristics of the patients between the modeling and validation groups were compared, and the differences showed no statistical significance (Table 1).

**Table 1:** Comparison of preoperative information between the modeling and validation groups

	Modeling group				Validation group	
	No. 10 + %	No. 10 - %		Total%	Total%	
	n = 31	n = 267	P	n = 298	n = 100	P
Age (years)	59.94 ± 9.25	60.49 ± 10.42	0.775	60.44 ± 10.29	61.74 ± 11.39	0.626
Gender			0.748			0.612
Male	23	205		228	74	
Female	8	62		70	26	
BMI (kg/m^2^)	22.60 ± 2.86	22.10 ± 2.69	0.33	22.15 ± 2.71	21.73 ± 2.51	0.51
Cross-sectional location			0.542			0.591
Greater curvature	5	23		28	7	
Lesser curvature	19	188		207	78	
Anterior wall	4	17		21	4	
Back wall	2	32		34	10	
Complete cycle	1	7		8	1	
Tumor size (cm)			<0.001			0.768
<5	2	133		135	47	
≥5	29	134		163	53	
Preoperative T stage			<0.001			0.876
With serosal invasion	20	64		84	29	
Without serosal invasion	11	203		214	71	
Preoperative N stage			<0.001			0.814
N0	6	153		159	52	
N+	25	114		139	48	
Accompanying diseases			0.443			0.208
Yes	11	77		88	23	
No	20	190		210	77	
Previous upper abdominal surgery			0.612			0.685
Yes	1	5		6	1	
No	30	262		292	99	
Preoperative Charlson score			0.861			0.466
'0	21	191		212	78	
1∼2	10	72		82	19	
≥3	0	4		4	3	
ASA			0.849			0.685
1	19	180		199	72	
2	12	76		88	25	
3	0	11		11	3	
Preoperative ALB	40.28 ± 3.06	39.96 ± 4.45	0.701	39.99 ± 4.33	38.91 ± 4.25	0.557
Preoperative HB	134.23 ± 17.04	125.83 ± 22.96	0.05	126.71 ± 22.54	120.48 ± 25.67	0.018
Terminal branches of the SpA			0.881			0.149
Concentrated type	22	186		208	62	
Distributed type	9	81		90	38	
Neoadjuvant chemotherapy			0.413			0.64
Yes	1	19		20	5	
No	30	248		278	95	

BMI: body mass index; SpA: splenic artery.

Table 2 summarizes the intraoperative and postoperative clinicopathological characteristics of the patients in the modeling group. The number of metastasizing LNs, blood loss volume (BLV) of splenic LNs and number of vascular clamps used for the splenic hilus and undifferentiated adenocarcinoma were significantly higher in patients with No. 10 LN metastasis than those in patients without No. 10 LN metastasis. A total of 44 (14.8%) patients showed postoperative complications. The general complications observed in patients with No. 10 LN metastasis were similar to those in patients without No. 10 LN metastasis (P = 0.447). However, abdominal chyle leakage in patients with No. 10 LN metastasis was significantly more prevalent than that in patients without No. 10 LN metastasis (P = 0.004) (Table 2).

**Table 2:** Comparison of the intraoperative and postoperative information between No. 10+ and No. 10- patients

	No. 10 + %	No. 10 - %	
	n = 31	n = 267	P
No. of positive LNs	23.29 ± 15.05	6.04 ± 8.21	<0.001
No. of retrieved LNs	44.32 ± 15.36	42.86 ± 15.46	0.618
No. of retrieved No. 10 LNs	3.35 ± 1.76	2.59 ± 2.18	0.061
BLV (mL) of splenic LNs	27.10 ± 22.64	19.69 ± 19.37	0.049
Operation time (min) for No. 10 LNs	24.26 ± 9.59	23.60 ± 9.02	0.704
No. of vascular clamps used for the splenic hilus	11.58 ± 4.39	10.04 ± 3.42	0.023
Spleen adhesion	10	85	0.962
Branch points of SpA			0.908
Division above the pancreas	30	251	
Division along pancreas	0	13	
Division between the hilar and pancreatic tail	1	3	
Type of SpA			0.396
Single branch SpA	4	14	
2-branched SpA	19	223	
3-branched SpA	8	30	
Polar artery of the spleen			
No. of SUPA	8	59	0.64
No. of SLPA	2	32	0.36
No. of SGA			0.691
<4	13	122	
≥4	18	145	
No. of PGA			0.006
0	16	74	
1	15	193	
Conversion			0.003
Yes	1	0	
No	30	267	
Vascular injury			0.212
Yes	17	115	
No	14	152	
Histology			0.009
Differentiated	9	144	
Undifferentiated	22	123	
Day of first flatus, d	4.13 ± 0.96	4.15 ± 1.08	0.918
Day of first fluid diet, d	4.74 ± 1.41	4.89 ± 1.95	0.687
Day of first semifluid diet, d	7.97 ± 1.85	8.39 ± 4.42	0.602
Postoperative hospital stay, d	12.81 ± 7.89	12.65 ± 6.68	0.915
General complications	6 (2.0)	38 (12.8)	0.447
Pulmonary infection	1 (0.34)	18 (6.0)	0.448
Wound infection	0	6 (2.0)	0.4
Lymphatic fistula	4 (1.3)	7 (2.3)	0.004
Anastomotic fistula	0	5 (1.7)	0.443
Intraperitoneal infection	2 (0.67)	10 (3.4)	0.469
Inflammatory intestinal obstruction	1 (0.34)	7 (2.3)	0.844
Anastomotic bleeding	0	2 (0.67)	0.629
Clavien Dingo classification			0.611
II	4 (1.3)	23	
IIIA	2 (0.67)	9 (3.8)	
IIIB	0	3 (1.0)	
IV	0	2 (0.67)	
V	0	1 (0.34)	

BLV: blood loss volume; SpA: splenic artery; SUPA: splenic upper polar artery; SLPA: splenic lower polar artery; SGA: short gastric artery; PGA: posterior gastric artery

### No. 10 lymph node metastasis and prognosis in the model development group

**Figure 1:**
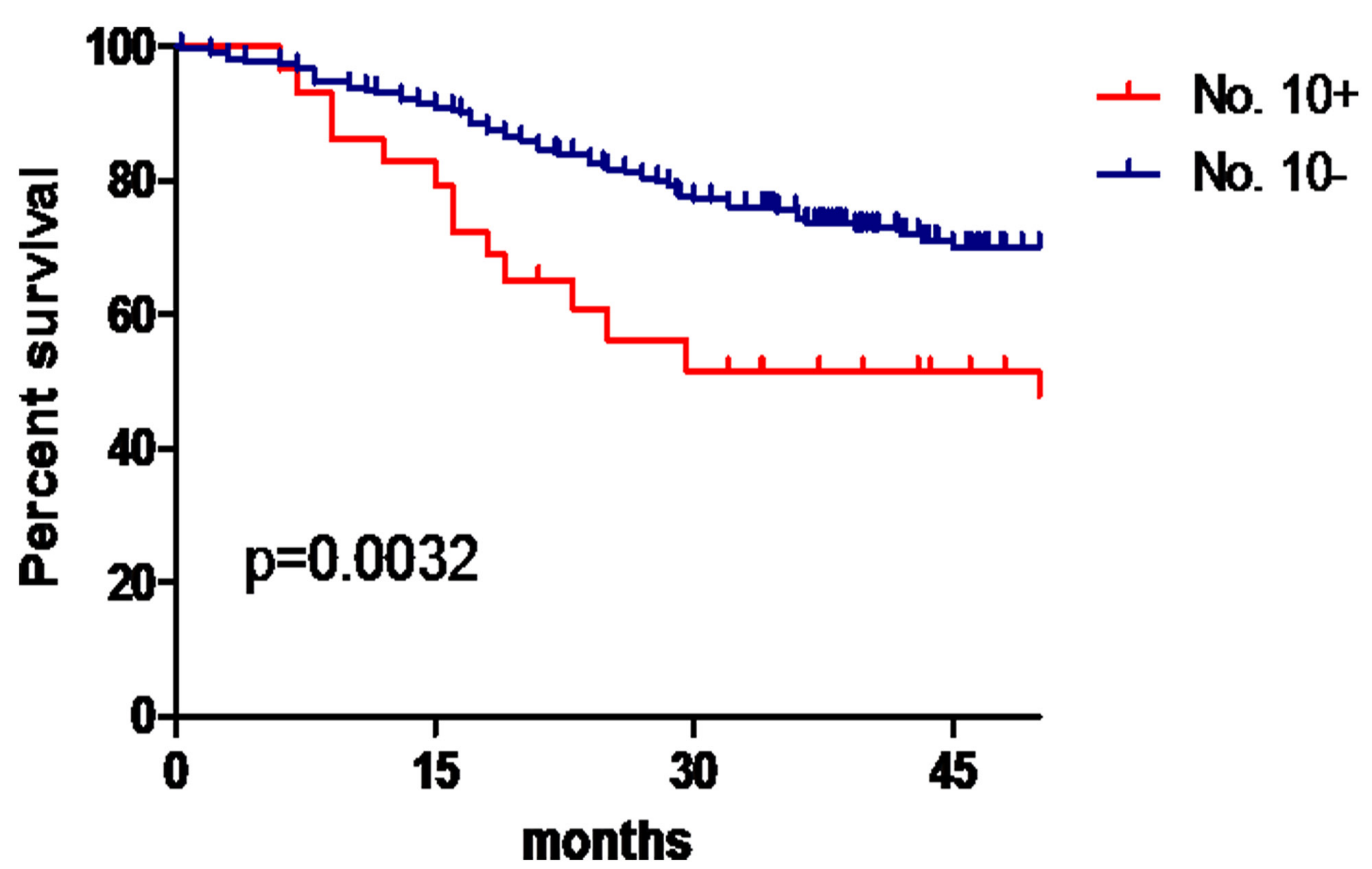
No. 10 LN metastasis and prognosis in the model development group

A total of 38 cases of No. 10 LN metastasis were identified in the model development group, yielding a transfer rate of 9.6. The median follow-up period was 33 (range, 18-66) months. The 3-year overall survival rate was 72.3%. The 3-year OS for patients with No. 10 LN metastasis was significantly shorter than that for patients without No. 10 LN metastasis (51.6% vs. 74.5%, P = 0.002) (Figure 1).

### Preoperative relative factor analysis associated with No. 10 LN metastasis

Table 3 shows the results of the univariate and multivariate analyses of the potential risk factors for patients with No. 10 LN metastasis. Three factors were associated with an increased risk of No. 10 LN metastasis, including preoperative tumor size, preoperative T stage, and preoperative N stage (P < 0.003). The results of the multivariate analysis identified preoperative tumor size (P = 0.006), preoperative T stage (P = 0.001), and preoperative N stage (P = 0.029) as adverse risk factors for No. 10 LN metastasis. We divided the cases into 2 groups according to the tumor size (≥5 cm, <5 cm). The No. 10 LN metastasis rate for each subgroup is shown in Table 4.

**Table 3:** Preoperative-related factors analysis of No. 10 LN metastasis

	Single factor analysis	Multiple factor analysis				Score
	p	B	OR	95%CI	p	
Tumor size (cm)	<0.001	2.094	8.114	1.831-35.952	0.006	2
<5						
≥5						
Preoperative T stage	<0.001	1.338	3.811	1.674-8.678	0.001	1
With serosal invasion						
Without serosal invasion						
Preoperative N stage	<0.001	1.082	2.951	1.114-7.815	0.029	1
N0						
N+						

**Table 4:** Combined preoperative T staging, tumor size and preoperative N staging assessment of No. 10 LN metastasis

Preoperative T stage	N0 (n,%)	N+ (n,%)
Tumor size (cm) ≥5		
With serosal invasion	3/41 (7.3)	15/43 (34.9)
Without serosal invasion	3/19 (15.8)	8/60 (13.3)
Tumor size (cm) <5		
With serosal invasion	0/11 (0)	2/11 (18.2)
Without serosal invasion	0/90 (0)	0/23 (0)

### Construction of the forecast model of No. 10 LN metastasis and degree of hazard grouping

**Figure 2:**
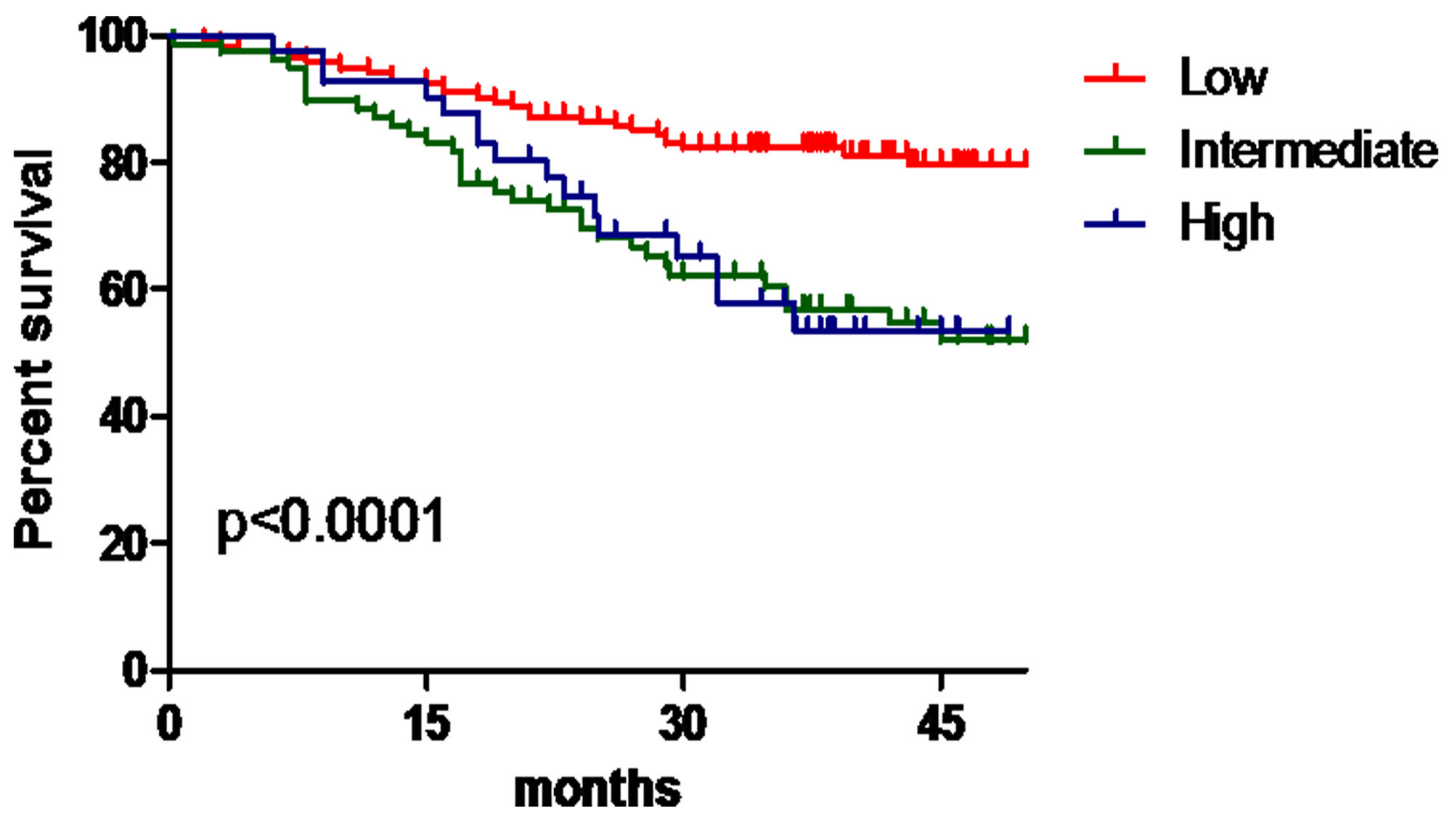
The OS for patients in the low-, intermediate- and high-risk groups

Despite differences in the regression coefficients, which ranged from 1.082 to 2.094 for No. 10 LN metastasis, for simplicity, 1 point was assigned for the preoperative T stage and preoperative N stage and 2 points were assigned for the tumor size. The resulting TNS (preoperative T stage, preoperative N stage, and tumor size) scores were obtained for No. 10 LN metastasis, and the patients in the modeling group were divided into three levels according to this scoring system: low risk (0-2 points, because the incidence rates of No. 10 LN metastasis among patients in 0 and 1 points was 0, 0-2 points were classified as low risk level), intermediate risk (3 points) and high risk (4 points). The distribution of patients according to the scoring system was 59.1% low risk; 26.5% intermediate risk; and 14.4% high risk. The incidence rates of No. 10 LN metastasis among patients in the low-, intermediate-, and high-risk categories were 2.8%, 13.9%, and 34.9%, respectively (χ^2^ = 28.60, P < 0.001). The relative risks of No. 10 LN metastasis in the intermediate- and high-risk groups compared with those of the low-risk group were 5.532 (95 %CI, 1.853-16.518, P = 0.002) and 18.321 (95 %CI, 6.171-54.392, P < 0.001), respectively (Table 5). The 3-year OS for patients in the low-risk category was significantly higher than that for patients in the intermediate- and high-risk groups (81.3% vs. 60.8% vs. 65.1%, P = 0.001) (Figure 2).

**Table 5:** Construction of the forecast model of No. 10 LN metastasis and the grouping degree of hazard

Risk group	Score	No. patients (n = 298 %)	No. patients (n %)	OR	95%CI	P
Low	0∼2	176	5	1	/	/
Intermediate	3	79	11	5.532	1.853-16.518	0.002
High	4	43	15	18.321	6.171-54.392	<0.001

### Discrimination

**Figure 3:**
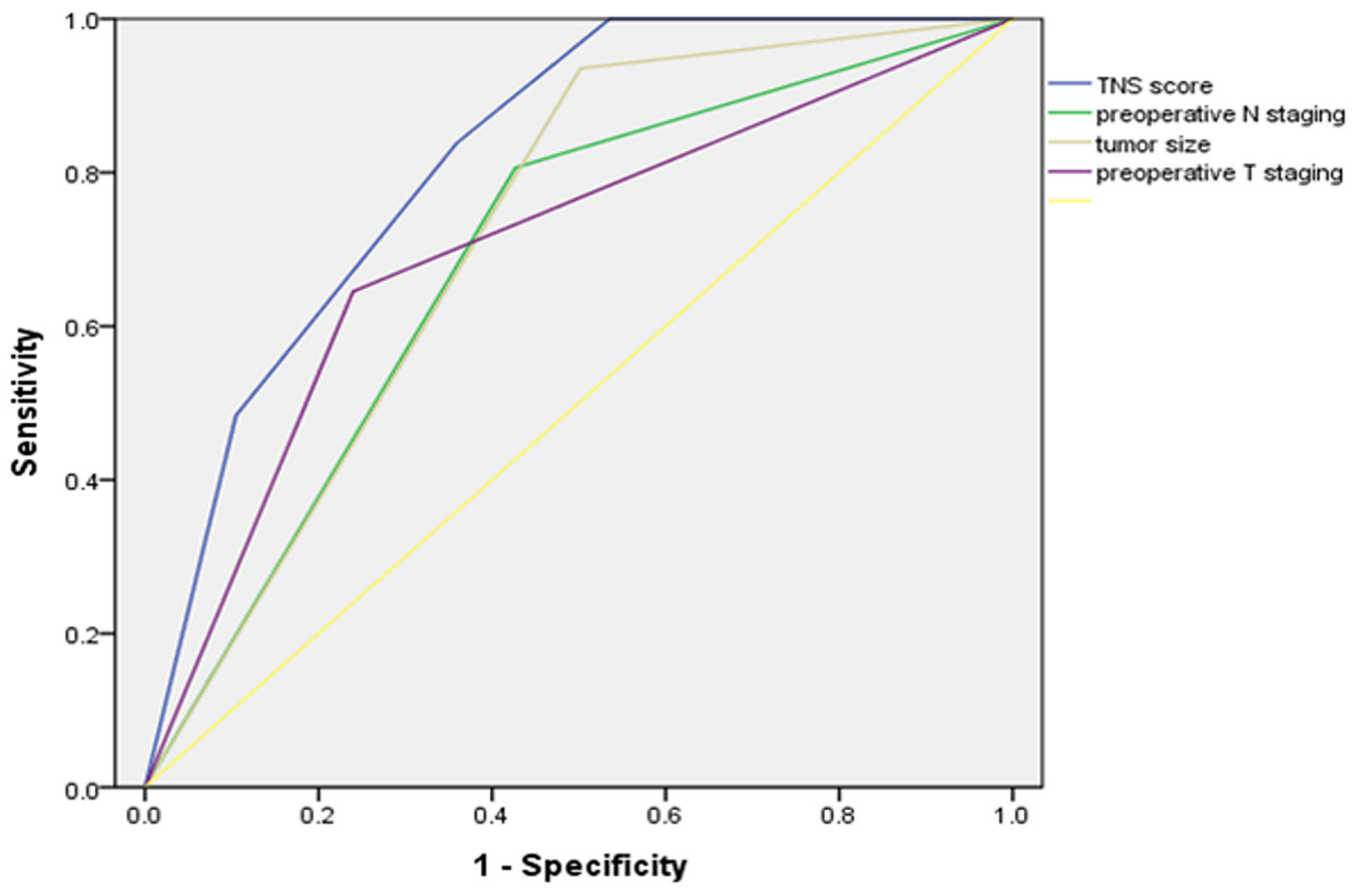
Receiver operating characteristic curves for prediction of TNS score for No. 10 LN metastasis compared with preoperative N staging, tumor size or the preoperative T staging in the development sets

The area under the ROC curve was 0.820 for the simplified TNS score for No. 10 LN metastasis. Compared with tumor size, preoperative N staging or the preoperative T staging, the TNS score more accurately predicts No.10 LN metastasis (Figure 3). The incidence rates observed in the validation set were compared to the predicted incidence rates to evaluate the model performance. The ratio of the expected to observed risk of No. 10 LN metastasis was 1.49, (χ^2^ = 0.84, P = 0.359), indicating good calibration. The ratios of the expected to observed risks for the low-, intermediate-, and high-risk categories in the validation set were 1.56, 1.08, and 2.09, respectively. There were no statistically significant differences among the groups (Table 6).

**Table 6:** Validation of the test model

Degree	Observed incidence rates of No. 10 LN metastasis (%)	Predicted incidence rates of No. 10 LN metastasis (%)	p
Low	1.8	2.8	0.66
Intermediate	12.9	13.9	0.902
High	16.7	34.9	0.36

## DISCUSSION

GC is one of the most common malignant tumors of the digestive system. Individualized effective treatment must be developed to enhance the postoperative survival rate of GC patients. LN metastasis is an important factor decreasing the prognosis of patients with GC, and thorough LN dissection significantly influences the success or failure of the surgery. Currently, the D2 LN dissection technique is considered the standard operation for advanced GC. No. 10 LN dissection is critical, and difficulties in D2 radical advanced upper GC have been reported. According to previous studies, the No. 10 LN metastasis rate is approximately 9.5% to 27.9% [1–3]. The results of the present study showed that the incidence of No. 10 LN metastasis was 9.55 in upper GC. Chikara et al. [13] reported that the 5-year OS of No. 10 LN metastasis-positive patients was significantly lower than that of No. 10 LN metastasis-negative patients (23.8% vs. 41.4%, P < 0.05), and these authors suggested that spleen regional LN metastasis has a significant effect on the prognosis of patients. Shin et al. [14] showed that the 5-year OS of No. 10 LN metastasis-positive patients was significantly lower than that of No. 10 LN metastasis-negative patients (11.04% vs. 51.57%, P < 0.001). Therefore, the dis-section of the No. 10 LN is necessary in advanced upper GC; otherwise, the radical excision of the tumor will be decreased. The concept of laparoscopic spleen-preserving SHLND is becoming increasingly accepted with the increasing application of laparoscopic GC radical surgery and advancements in surgical instruments. However, whether all cases of advanced upper GC should be treated using this laparoscopic spleen-preserving SHLND technique has not yet been determined. Therefore, how to predict splenic hilar LN metastasis and characterize the indications for SHLND has become a topic of considerable interest in the research community. However, previous studies analyzing the risk factors for No. 10 LN metastasis were confined to postoperative-related factors and thus were not applicable for preoperative prediction. To our knowledge, the present study is the first preoperative retrospective study investigating the factors related to No. 10 LN metastasis in upper GC.

Based on previous studies of splenic hilar LN metastasis, we established that No. 10 LN metastasis is depended on primary tumor size, depth of invasion, general classification, and tumor cell type. The No. 10 LN metastasis rate of upper GC is significantly higher than that of lower GC [15]. In the present study, a multivariate analysis was used to examine preoperative tumor size, preoperative T stage, and preoperative N stage as adverse risk factors for No. 10 LN metastasis. An analysis of 219 patients who underwent SHLND showed that the depth of invasion was closely correlated with No. 10 LN metastasis [16]. The likelihood of No. 10 LN metastasis in patients with advanced GC was increased in patients with tumors penetrating the subserosa or muscularis, with more than 7 macroscopic LN metastases. Koga et al. [17] reported that No. 10 LN metastasis frequently appeared in Borrmann type IV cancer or when the primary tumor involved the serosa or entire stomach. We propose that with tumor development, the growth and invasion depth of the tumor continuously progresses, and eventually, the tumor penetrates through the muscle layer and infiltrates the placenta percreta layer because the serous layer contains an abundant capillary lymphatic network; thus, the incidence of No. 10 LN metastasis increases significantly. Moreover, GC patients with other LN metastases are closely related to No. 10 LN metastasis. The increased LN metastases and extensive transfer of the patients of preoperative staging of cN+, increases the probability of No. 10 LN metastasis. Due to the invasion of the metastatic LNs, the local anatomic structure is more complex, the intraoperative lymphadenectomy is more difficult and risky, thus more blood loss is caused by splenic vessel injury during surgery in patients with No. 10 LN metastasis than those in patients without No. 10 LN metastasis.Therefore, even if there is no difference in No. of retrieved No. 10 LNs between the two groups, patients with No. 10 LN metastasis revealed more blood loss and need more vascular clamps than those without No. 10 LN metastasis.

**Figure 4:**
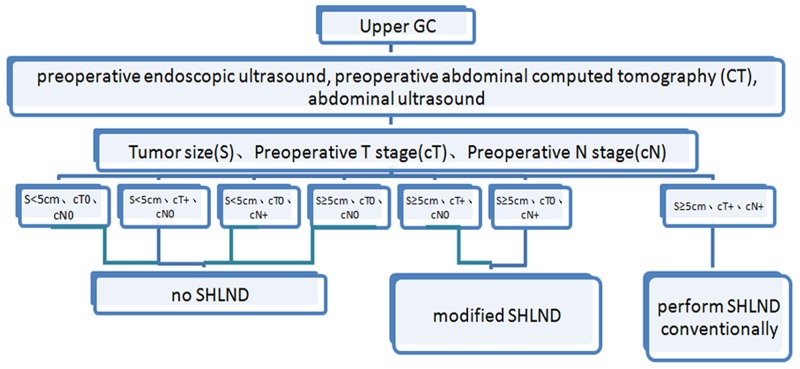
Proposed algorithm for the medical evidence for SHLND indications

According to the prediction for the risk of No. 10 LN metastasis, in a previous study, we demonstrated that prediction systems, including factors, such as the tumor infiltration depth and tumor transverse position, with No. 7 and No. 11 LN metastasis provide a better evaluation of No. 10 LN metastasis [15]. However, the guidelines for the preoperative significance are not strong considering the postoperative parameters. This study constructed a TNS scoring system to predict No. 10 LN metastasis and classify patients into three categories: low, intermediate, and high risk. This scoring system identified a high-risk group, which has a 12.3-fold greater risk of No. 10 LN metastasis than the lowest-risk group, and this difference was statistically significant. Further analysis indicated that the area under the ROC curve was 0.820 for the simplified TNS score, with a discriminant ability similar to the logistic regression model, and in the validation group, there was no statistical significance between the observed and predicted incidence rates of No. 10 LN metastasis for the TNS scoring system. In the present study, we evaluated the related factors as preoperative readily available parameters for the simple and effective prediction of the risk of No. 10 LN metastasis and the preoperative discrimination of high-risk groups prone to transfer from low-risk groups, which has important significance for improving the prognosis of patients with upper GC by performing more corresponding, positive and effective targeted operation schemes. Therefore, in combination with the results of the present study, we propose that SHLND is not necessary for low-risk patients, whereas high-risk patients require regular treatments through SHLND. For patients with moderate risk, showing a 13.9% risk of No. 10 LN metastasis, we recommend SHLND to obtain a better radical cure effect (Figure 4).

Some shortcomings of this study should be noted. These results were based on clinical data obtained from an eastern country at a single institution. Eastern countries have higher GC morbidity and more advanced-stage GC patients than western countries. Moreover, the average BMIs in eastern countries are lower than those in western countries. The results showed there is no significant difference in No. 10 LN metastasis between the patients with and without neoadjuvant chemotherapy, regardless of the modeling or validation group, which proving that the neoadjuvant chemotherapy didn't change the original status of No. 10 LNs. Therefore, a prospective multiple-center study with a large population would help validate this scoring system in a sample of patients from both eastern and western countries.

## MATERIALS AND METHODS

### Materials

This study presents a retrospective analysis of a prospectively collected database of upper- or middle-third GC patients treated with laparoscopic spleen-preserving SHLND in the Department of Gastric Surgery of Fujian Medical University Union Hospital, Fuzhou, China, between January 2011 and December 2014. The following inclusion criteria were used: (1) histologically confirmed primary adenocarcinoma in the upper- or middle-third stomach; (2) no evidence of tumor invasion in adjacent organs (pancreas, spleen, liver, or transverse colon), enlargement or integration of the para-aortic or splenic hilar LNs, or distant metastasis demonstrated by preoperative abdominal computed tomography (CT), abdominal ultrasound or endoscopic ultrasound; and (3) a total gastrectomy plus D2 lymphadenectomy with curative R0 resection based on the postoperative pathological diagnosis. The exclusion criteria were patients with T4b tumors; incomplete clinicopathological data; intraoperative evidence of peritoneal dissemination or distant metastasis; or gastric stump carcinoma. A total of 398 patients, including 302 males and 96 females with a mean age of 60.76 ± 10.58 years, were included in the present study. The preoperative size, location, T stage (with or without the presence of serosal invasion) and N stage (with or without LN metastasis) of the neoplasm were assessed in all patients via upper digestive endoscopy with biopsy, chest X-ray, total abdominal ultrasound, and abdominopelvic CT scan. Preoperative comorbidities were described according to the classification system of the American Society of Anesthesiologists [6]. CT scans and multi-slice spiral CT angiography were performed to preoperatively assess the splenic vascular anatomy. BLV(mL) of splenic LNs was estimated according to the number of the gauze blocks and the trip attraction of aspirator, A piece of “two by two” is equivalent to 4 ml.The patient demographics, underlying diseases, clinicopathology, and preoperative and postoperative monitoring data were recorded in a clinical data system for GC surgery. The type of surgical resection and extent of LN dissection were selected according to the Japanese gastric cancer treatment guidelines [7]. The resected specimens were histopathologically examined and staged according to the 7th edition of the UICC TNM classification [8]. According to the reported references [9–11], data were randomly divided into two subsets using SPSS version 18.0 (SPSS, Chicago, IL, USA) to create a 75/25 split, with one subset used for model development and the other used for validation testing.

The research proposal was reviewed by the Research Ethical Committee at the university, and all procedures were performed after obtaining written informed consent from the patients following an explanation of the surgical and oncological risks.

### Follow-up

Trained investigators performed the postoperative follow-ups through mailings, telephone calls, home visits or outpatient services. The majority of patients routinely underwent physical examinations, laboratory testing (including CA19-9, CA72-4, and CEA levels), chest radiography, abdominal US or CT, and an annual endoscopic examination. Overall survival (OS) was calculated from the day of surgery until death or until the final follow-up date of June 2016, whichever occurred first.

### Statistical analysis

The continuous data are reported as the means ± SD, and the differences between the groups were analyzed using t-tests. The categorical data are presented as the proportion and percentage, analyzed using the chi-square test or Fisher’s exact test. The variables in the models reaching P < 0.05 in the univariate analysis were subsequently included in a multivariate binary logistic regression model. The variables that remained significant in the multivariate analysis were used to construct a scoring system to classify patients into groups based on their risk for laparoscopic hemostasis. The results of the multivariate analyses are expressed as odds ratios (ORs) with corresponding 95% confidence intervals (95%CIs). A goodness-of-fit test was conducted to assess how well the model could discriminate between patients with and without No. 10 LN metastasis. Receiver operating characteristic (ROC) and area under the curve (AUC) analyses were used to determine the adequacy of the prediction models. Values of 0.7 and higher were considered clinically significant [12]. The model calibration, or the degree to which the observed outcomes were similar to the outcomes predicted by the model across patients, was examined by comparing the observed averages with the predicted averages within each subgroup, arranged in increasing order of patient risk. Values of P < 0.05 were considered statistically significant. The statistical analyses were performed using SPSS version 18.0 (SPSS, Chicago, IL, USA).
